# The Role of Problematic Smartphone Uses and Psychological Distress in the Relationship Between Sleep Quality and Disordered Eating Behaviors Among Chinese College Students

**DOI:** 10.3389/fpsyt.2021.793506

**Published:** 2021-12-13

**Authors:** Ruipeng Wu, Lan Guo, Hao Rong, Jingming Shi, Wenyan Li, Minxia Zhu, Yongjun He, Wanxin Wang, Ciyong Lu

**Affiliations:** ^1^Department of Medical Statistics and Epidemiology, School of Public Health, Sun Yat-sen University, Guangzhou, China; ^2^Guangdong Provincial Key Laboratory of Food, Nutrition and Health, Sun Yat-sen University, Guangzhou, China; ^3^Key Laboratory of High Altitude Hypoxia Environment and Life Health, School of Medicine, Xizang Minzu University, Xianyang, China; ^4^Key Laboratory for Molecular Genetic Mechanisms and Intervention Research on High Altitude Disease of Tibet Autonomous Region, School of Medicine, Xizang Minzu University, Xianyang, China

**Keywords:** disordered eating behaviors, sleep quality, problematic smartphone use, anxiety symptoms, depressive symptoms

## Abstract

**Background:** Sleep problems and eating disorders (EDs) are both serious public health concerns often seen in young adults. Yet, the underlying mechanisms for such associations are largely unknown. This study aims to examine potential serial multiple mediation effects of problematic smartphone use (PSU) and psychological distress (i.e., depressive and anxiety symptoms) in the relationship between sleep quality and disordered eating behaviors/attitudes (DEBs).

**Methods:** A total of 4,325 students from two Tibet universities in China (2,657 females and 1,668 males) completed an online survey that included the following measurements: Eating Attitude Test-26 for disordered eating behaviors/attitudes, the Chinese Version of Pittsburgh Sleep Quality Index (CPSQI), Smartphone Addiction Scale—Short Version (SAS-SV) for problematic smartphone use, Patient Health Questionnaire-9 (PHQ-9) and Generalized Anxiety Disorder-7 (GAD-7) for psychological distress.

**Results:** While the direct path linking sleep quality and DEBs was not found to be significant (Standardized β = 0.006, 95% CI = −0.0667~0.0970), both PSU (Standardized β = 0.016, 95% CI = 0.0256~0.0591) and anxiety symptoms (Standardized β = 0.014, 95% CI = 0.0203~0.0526) may mediate a link between sleep quality and DEBs; serial multiple mediation analysis revealed that a serial indirect pathway of “sleep quality -> PSU -> anxiety symptoms -> DEBs” existed(Standardized β = 0.001, 95% CI = 0.0002~0.0012). Similarly, while the direct path linking sleep quality and DEBs was not found to be significant (Standardized β = 0.006, 95% CI = −0.0667~0.0970), both PSU (Standardized β = 0.020, 95% CI = 0.0337~0.0692) and depressive symptoms (Standardized β = 0.015, 95% CI = 0.0139~0.0652) may mediate a link between sleep quality and DEBs; serial multiple mediation analysis revealed that a serial indirect pathway of “sleep quality -> PSU -> depressive symptoms -> DEBs” existed (Standardized β = 0.001, 95% CI = 0.0006~0.0038).

**Conclusions:** Psychological and behavioral factors may comprehensively work together, leading to flow-on effects from sleep problems to disordered eating behaviors among university students. Appropriate interventions that target problematic smartphone use could thus potentially reduce anxiety and depression levels, which in turn will provide a buffer against the negative impact of poor sleep quality on eating disorder symptoms.

## Introduction

Eating disorders (EDs) are serious psychiatric disorders with core features such as disturbance in body image, extreme eating behaviors, and weight control (1, 2). The lifetime prevalence rate of EDs is 2–8% in the US (3), and 20–20.6% of the college students were at risk of an eating disorder in some South-East Asian countries (4). Recent studies show that the prevalence of EDs in China is increasing (5). Tong et al., found a comparable prevalence of EDs in female university students (3.53% for binge-eating disorder, 2.98% for bulimia nervosa, and 1.05% for anorexia nervosa) to that of their western counterparts (6, 7). EDs are associated with a variety of adverse outcomes, which seriously affect people's quality of life (8–12). However, research on eating disorders in China has not attracted enough attention.

Many factors may be related to increased risks of EDs, and several are especially prevalent for students in the stage of emerging adulthood (18–26 years old). Sleep abnormality may have an effect through impacting physical and mental well-being (13–16), along with fluctuations of several hormones such as cortisol, leptin, melanocortin (1, 17–19). In addition, problematic smartphone use (PSU) and psychological distress such as depression may also affect both sleep quality and EDs. The interconnectedness and bidirectional relationships of these physiological, psychological, and behavioral pathways have not been fully understood, thus the goal of this current study was to clarify the pathways that mediate these factors.

PSU has been reported to have a strong correlation with sleep quality where students' sleep quality worsens with increasing mobile phone addiction levels, and this relationship may be bidirectional (20–23). PSU is also related to EDs (24–27). Taken together, PSU could play a mediating role between sleep quality and eating disorders. Similarly, psychological distress including anxiety and depression has also been reported to be associated with sleep disorders (28–31) and EDs (12, 32–37) independently. And, the proposed mediating roles of anxiety/depression between sleep disorders and ED symptomology have been repeatedly shown in previous studies involving different samples (e.g., Inpatient, children, college women, and community adults) (9, 15, 36, 37).

There were also links reported between PSU and psychological distress (38, 39), where Sangmin Jun et al. found that the relationship between mobile phone addiction and depressive symptoms may present a vicious circle using a longitudinal data (38). Moreover, smartphone addiction was found to mediate the relationship between negative affect and sleep quality among Chinese university students (23).

**Figure 1:**
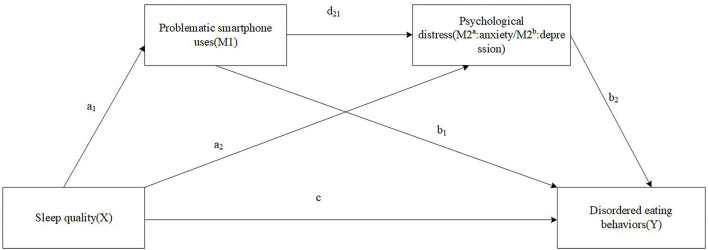
The proposed serial multiple mediation of problematic smartphone uses and psychological distress between sleep quality and disordered eating behaviors.

Given the correlations stated above are established based on studies conducted across a wide range of countries mostly focusing on western cultures, the current study aims to extend upon these researches to provide valuable data on students in Tibet that possesses unique culture and diet. The association between sleep quality and disordered eating was investigated by examining serial multiple mediations linking sleep quality to EDs through PSU and psychological distress (i.e., depressive and anxiety) in Chinese Tibetan college students. Given that EDs require clinical diagnosis, disordered eating behaviors/attitudes (DEBs) were used, which refer to behaviors that deviate from normal but have not yet met the diagnostic criteria for eating disorder (40). We hypothesize that sleep quality would be related to DEBs and that PSU and depressive/anxiety would independently and in series mediate the association between sleep quality and DEBs (Figure 1). Through clarifying a pathway, our results may help inform the development of effective intervention and prevention strategies targeting young adults' DEBs.

## Methods

### Participants

A cross-sectional study was conducted from June 2021 to July 2021 by cluster convenience sampling from two universities in Tibet of China. The survey was completed on the online platform (Wenjuanxing) (41). About 15 student cadres from different colleges are recruited and trained as research assistants. Each research assistant sends a pre-made link containing the questionnaire content to the class's WeChat group or QQ group. Each IP was set to accept only one response. Participants were informed in advance of the purpose of the survey and were voluntarily enrolled. All information provided by participants was confidential and anonymous. A total of 4,885 respondents completed and submitted the survey. Based on the method recommended by Greszki et al. 507 individuals whose response times were very short were eliminated (42). In addition, 53 cases with incomplete information or were answered identically for each question were also deleted. Overall, 4,325 subjects were included in the analysis with an effective response rate of 88.5%.

### Measures

#### Demographic and Clinical Characteristics

The demographic and clinical characteristics included sex (males = 1, females =2), age, ethnicity (Han =1, Tibetan = 2, others = 3), household socioeconomic status (HSS), smoking, drinking and body mass index (BMI) were collected. HSS was measured by asking students' perceptions of their current family economic situation (Response categories: excellent or very good = 1, good =2, and fair or poor =3). BMI was calculated based on students' self-reported height and weight. Based on the BMI standards for Chinese adults (43), the subjects were classified as underweight (BMI < 18.5 kg/m^2^), normal weight (18.5–23.9), overweight (24–27.9) and obese (>28). Students that smoked or drank alcohol at least once in the past 30 days were classified as current smokers or drinkers, respectively (44, 45).

#### Disordered Eating Behaviors/Attitudes

Eating Attitude Test-26 (EAT-26) is a self-administrated questionnaire that evaluates disordered eating behaviors/attitudes (46). The questionnaire consists of 26 items, which mainly involve diet-related attitudes, beliefs and behaviors, and appearance perception. Each item of EAT-26 is scored on a 6-point Likert scale ranging from 0 (never) to 5 (always). The EAT-26 consists of three sub scales: diet, bulimia and food preoccupation, and oral control. After converting the 6-point Likert score into a 4-point format, the total score (ranges from 0 to 78) is calculated by summarizing all items. A higher EAT-26 total score indicates more eating disorder symptoms. The Chinese version of the EAT-26 demonstrated good internal consistency, test-retest reliability and convergent validity (47). The Cronbach's α of the EAT-26 in the present study was 0.855.

#### Sleep Quality

Sleep quality over the past month was measured by the self-reported Chinese Version of the Pittsburgh Sleep Quality Index (CPSQI) (48). It contains questions regarding 7 sleep components (each scored on a 0–3 scale): subjective sleep quality, sleep latency, sleep duration, habitual sleep efficiency, sleep disturbance, use of sleep medications, and daytime dysfunction. The global score, which is the cumulative score of seven components, can range from 0 to 21. A higher score indicates poorer sleep quality. The CPSQI has good reliability and validity (48).

#### Problematic Smartphone Use

Problematic smartphone use severity was assessed by the Smartphone Addiction Scale-short Version (SAS-SV) (49), which is the shortened version of the original SAS (50). The SAS-SV consists of 10 items with response options from “Strongly disagree = 0” to “Strongly agree = 6.” The total score of the SAS-SV ranged from 10 to 60, with a higher score representing higher risk of PSU. The Chinese scale version of SAS-SV has a good internal consistency (49). Cronbach's coefficient in our sample was 0.912tba1.

#### Psychological Distress

Depressive symptoms were measured by Patient Health Questionnaire-9 (PHQ-9), which is a self-report questionnaire consisting of nine items matching the Diagnostic and Statistics Manual of Mental Disorders-Fifth Edition criteria of major depression (51). Each item is used to evaluate feelings in the past 2 weeks. Response options ranged from “not at all = 0” to “nearly every day = 3.” The total sum of PHQ-9 scores ranges from zero to 27. The Cronbach's alpha coefficient in this study was 0.920.

Symptoms of anxiety were assessed by using the Generalized Anxiety Disorder-7 (GAD-7) (52). The seven items reflect the frequency of the seven core symptoms in the past 2 weeks, using a 4-point Likert rating scale for duration assessment “not at all = 0” to “almost every day = 3.” The total score of GAD-7 ranged from 0 to 21. Higher scores represent higher severity for anxiety. In this study, Cronbach's coefficient of the GAD-7 was 0.937.

### Statistical Analysis

Data analyses were carried out in SPSS version 23.0 for Windows (IBM Corp., Armonk, New York, USA). First, descriptive analyses were used to describe the sample characteristics across sex; *t*-tests or chi-square tests were used to compare between groups. Second, the bivariate relationships of the studied variables were examined using Pearson's bivariate correlations. Third, the serial multiple mediating models were tested using the SPSS PROCESS macro version 3.3 developed by Preacher and Hayes, with model 6 and 10,000 bootstrapping samples (53). A significant effect was inferred statistically if the 95% bootstrap confidence interval (CI) did not include 0. Two serial multiple mediation models of a and b were analyzed to examine PSU(M1) and psychological distress (M2^a^ as anxiety and M2^b^ as depression, respectively) as serial mediators in the relationship between sleep quality (X) and DEBs (Y) (as shown in Figure 1). The total indirect effect of each model included three specific indirect effects as follows: (1) through PSU (a_1_b_1_), through psychological distress (a_2_b_2_), and through PSU and psychological distress in serial (a_1_d_21_b_2_). All the models controlled for sex, age, ethnicity, HSS, BMI, smoking, and drinking. Model (a) controlled for depression in particular, and model (b) controlled for anxiety. Missing data of relevant variables was <2% and eliminated in the serial multiple mediating analyses. *P*-value < 0.05 was considered statistically significant (2-sided tests).

## Results

### Demographics and Anterior Analyses

The data on the distribution of basic demographic information and some variables of participants according to sex are shown in Table 1. Of the enrolled students, 38.6% (1,668) were males, 61.4% (2,657) were females, with an overall mean age of 19.9 (SD: ± 1.3) years. The proportions of Tibetan and Han students were 57.1% (2,470) and 40.3% (1,742), respectively. 69.0% (3,786) of students reported good and average household socioeconomic status. Underweight, overweight, and obese were 16.4% (709), 11.7 (506), and 5.0% (217), respectively. Approximately 20.6% (890) students admitted to smoking, and 53.4% (2,308) reported drinking. The mean scores of CPSQI, SAS-ST, GAD-7, PHQ-9, EAT-26 were significantly higher in female than male students (*p* < 0.001).

**Table 1:** Participant characteristics stratified by sex.

**Variable**	**Male, *n* (%)**	**Female, *n* (%)**	**Total, *n* (%)**	***p-*value^**#**^**
**Total**	1,668 (38.6)	2,657 (61.4)	4,325 (100)	
**Age (year)**	20.1 (1.6)	19.8 (1.1)	19.9 (1.3)	<0.001
**Ethnicity**				
Han	773 (46.3)	970 (36.5)	1,743 (40.3)	<0.001
Tibetan	849 (50.9)	1,621 (61.0)	2,470 (57.1)	
Others	46 (2.8)	66 (2.5)	112 (2.6)	
**HSS**				
Good	267 (16.0)	423 (15.9)	690 (16.0)	<0.001
Average	799 (47.9)	1,497 (56.3)	2,296 (53.0)	
Poor	602 (36.1)	737 (27.7)	1,339 (31.0)	
**BMI**				
Underweight	189 (11.4)	520 (19.6)	709 (16.4)	<0.001
Normal weight	1,092 (65.6)	1,793 (67.6)	2,885 (66.8)	
Overweight	258 (15.5)	248 (9.3)	506 (11.7)	
Obese	125 (7.5)	92 (3.5)	217 (5.0)	
**Smoking**				
No	911 (54.6)	2,524 (95.0)	3,435 (79.4)	<0.001
Yes	757 (45.4)	133 (5.0)	890 (20.6)	
**Drinking**				
No	502 (30.1)	1,515 (57.0)	2,017 (46.6)	<0.001
Yes	1,166 (69.9)	1,142 (43.0)	2,308 (53.4)	
**CPSQI score**	5.4 (2.9)	5.6 (2.7)	5.5 (2.8)	0.013
**SAS-ST score**	33.4 (10.1)	35.6 (9.1)	34.8 (9.6)	<0.001
**GAD-7 score**	10.9 (4.3)	11.7 (4.4)	11.4 (4.4)	<0.001
**PHQ-9 score**	13.8 (4.9)	14.5 (4.9)	14.2 (4.9)	<0.001
**EAT-26 score**	5.7 (6.4)	7.5 (7.3)	6.8 (6.9)	<0.001

*HSS, household socioeconomic status; BMI, body mass index; CPSQI, Chinese Version of the Pittsburgh Sleep Quality Index; SAS-ST, smartphone addiction scale-short version; GAD-7, Generalized Anxiety Disorder-7; PHQ-9, Patient Health Questionnaire-9; EAT-26, Eating Attitude Test-26*.

# *The chi-square test was used for categorical variables, and the t test was used for age, CPSQI, SAS-ST, GAD-7, PHQ-9 and EAT-26 scores*.

### Correlational Analysis

Sleep quality were positively associated with PSU (*r* = 0.248, *p* < 0.001), anxiety (*r* = 0.497, *p* < 0.001), depression (*r* = 0.537, *p* < 0.001), and DEBs (*r* = 0.187, *p* < 0.001). PSU were positively associated with anxiety (*r* = 0.296, *p* < 0.001), depression (*r* = 0.319, *p* < 0.001), and DEBs (*r* = 0.250, *p* < 0.001). Depression was positively associated with anxiety (*r* = 0.806, *p* < 0.001) and DEBs (*r* = 0.293, *p* < 0.001). Anxiety was positively associated with DEBs (*r* = 0.304, *p* < 0.001) (as shown in Table 2).

**Table 2:** Intercorrelations among study variables.

**Variable**	**1**	**2**	**3**	**4**	**5**
1. **Sleep quality**	1				
2. **PSU**	0.248^***^	1			
3. **Depression**	0.537^***^	0.319^***^	1		
4. **Anxiety**	0.497^***^	0.296^***^	0.806^***^	1	
5. **DEBs**	0.187^***^	0.250^***^	0.293^***^	0.304^***^	1

*** *p < 0.001*.

### Serial Multiple Mediating Analysis

**Figure 2:**
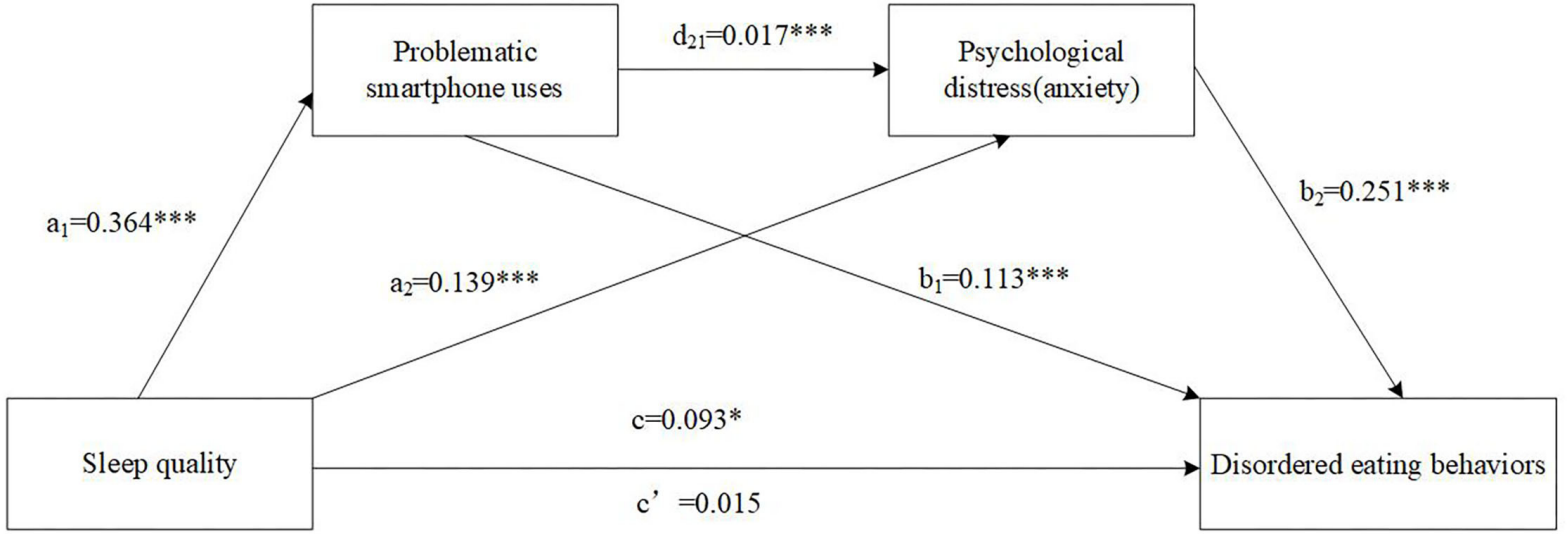
The serial multiple mediation of problematic smartphone uses and anxiety between sleep quality and disordered eating behaviors. Path coefficients are shown in unstandardized regression coefficient, c = total effect, c′ = direct effect. The covariates were sex, age, ethnicity, HSS, BMI, smoking, drinking and depression. ***p < 0.001, *p < 0.05.

Results of the serial multiple mediation model (a) including sleep quality, PSU, anxiety, and DEBs are displayed in Figure 2 and Table 3. The association between poor sleep quality and DEBs was statistically significant (total effect c: Standardized β = 0.037, 95% CI = 0.0104~0.1751), and a total of 15.7% of the variance was explained by the combined contribution of sleep quality and covariates. The specific indirect effect through PSU was significant (a_1_b_1_: Standardized β = 0.016, 95% CI = 0.0256~0.0591). The specific indirect effect through anxiety was significant (a_2_b_2_: Standardized β = 0.014, 95% CI = 0.0203~0.0526). Finally, the significant indirect effect of sleep quality on DEBs through both PSU and anxiety (a_1_d_21_b_2_: Standardized β = 0.001, 95% CI = 0.0002~0.0012) was tested. However, the direct effect of poor sleep quality on DEBs was not statistically significant (direct effect c': Standardized β = 0.006, 95% CI = −0.0667~0.0970).

**Table 3:** Results of the serial mediation models of PSU and psychological distress (i.e., depression and anxiety symptoms) on the relationship between sleep quality and DEBs.

**Path**	***B***	**β**	**SE**	**95%CI**	
				**Lower**	**Upper**
**Model** ^**a**^					
Total effect	0.093	0.037	0.042	0.0104	0.1751
Direct effect	0.015	0.006	0.042	0.0667	0.0970
Total indirect effect	0.078	0.031	0.013	0.0549	0.1053
Sleep quality -> PSU -> DEBs:	0.041	0.016	0.009	0.0256	0.0591
Sleep quality -> Anxiety -> DEBs:	0.035	0.014	0.008	0.0203	0.0526
Sleep quality -> PSU -> Anxiety -> DEBs:	0.002	0.001	0.001	0.0006	0.0030
**Model** ^**b**^					
Total effect	0.106	0.042	0.041	0.0264	0.1864
Direct effect	0.015	0.006	0.042	0.0667	0.0970
Total indirect effect	0.091	0.036	0.016	0.0613	0.1221
Sleep quality -> PSU -> DEBs:	0.051	0.021	0.009	0.0337	0.0692
Sleep quality -> Depression -> DEBs:	0.039	0.015	0.013	0.0139	0.0652
Sleep quality -> PSU -> Depression -> DEBs:	0.002	0.001	0.001	0.0006	0.0038

*B, non-standardized regression coefficient; β, standardized regression coefficient; SE, standard error; CI, confidence interval*.

*All models controlled for sex, age, ethnicity, HSS, BMI, smoking and drinking, with model*

a *controlled depression and model*

b *anxiety additionally*.

**Figure 3:**
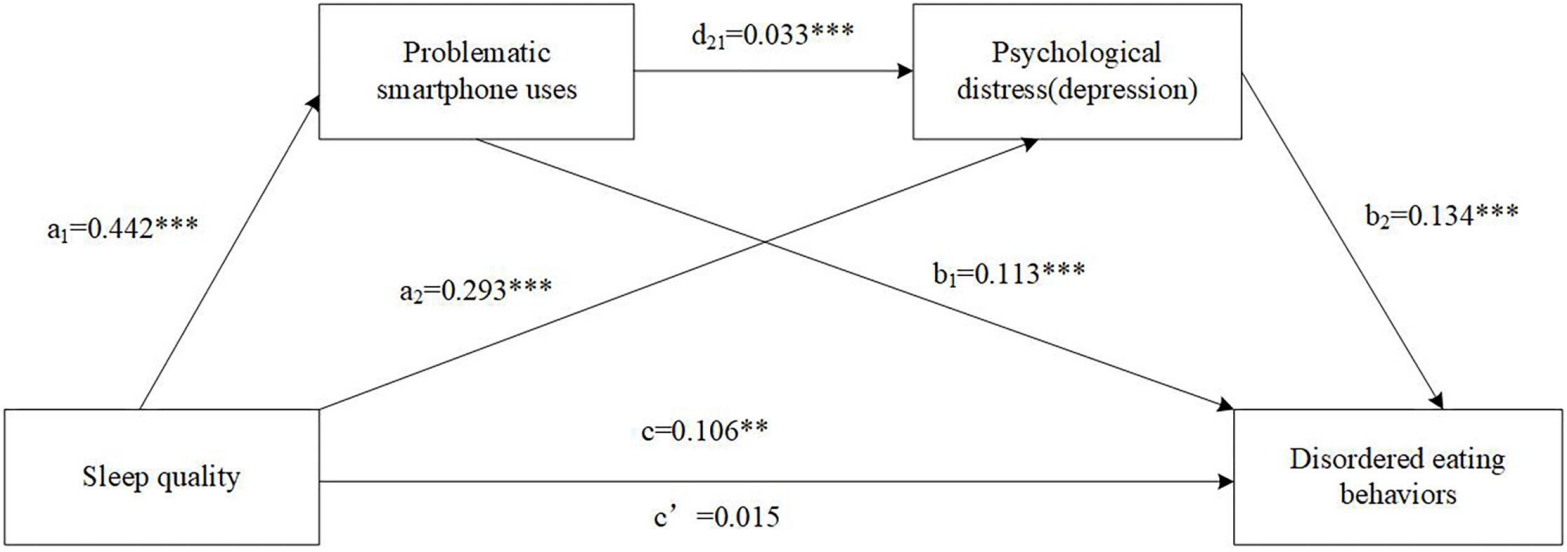
The serial multiple mediation of problematic smartphone uses and depression between sleep quality and disordered eating behaviors. Path coefficients are shown in unstandardized regression coefficient, c = total effect, c′ = direct effect. The covariates were sex, age, ethnicity, HSS, BMI, smoking, drinking and anxiety. ***p < 0.001, **p < 0.01.

Results of the serial multiple mediation model (b) including sleep quality, PSU, depression, and DEBs are displayed in Figure 3 and Table 3. The association between poor sleep quality and DEBs was statistically significant (total effect c: Standardized β = 0.042, 95% CI = 0.0264~0.1864), and a total of 13.9% of the variance was explained by the combined contribution of sleep quality and covariates. The specific indirect effect through PSU was significant (a_1_b_1_: Standardized β = 0.020, 95% CI = 0.0337~0.0692). The specific indirect effect through depression was significant (a_2_b_2_: Standardized β = 0.015, 95% CI = 0.0139~0.0652). Finally, the significant indirect effect of sleep quality on DEBs through both PSU and depression (a_1_d_21_b_2_: Standardized β = 0.001, 95% CI = 0.0006~0.0038) was tested. However, the direct effect of poor sleep quality on DEBs was not statistically significant (direct effect c': Standardized β = 0.006, 95% CI = −0.0667~0.0970).

## Discussion

The present study investigated the relationships among sleep quality, PSU, psychological distress and DEBs based on the demographic of Chinese Tibet university students. Poor sleep quality was found to be positively associated with DEBs. Results of serial multiple mediation analyses indicated that PSU and psychological distress (i.e., anxiety and depression) could fully mediate the relationship between sleep quality and DEBs. Multiple indirect pathways from sleep quality to eating disorder symptoms were revealed. First, PSU mediate the relationship between sleep quality and DEBs. Second, psychological distress (i.e., anxiety and depression) mediated the relationship between sleep quality and DEBs. Third, PSU and psychological distress (i.e., anxiety and depression) jointly played a serial mediating role in the relationship between sleep quality and DEBs.

Previously, despite the individual links established between sleep problems, problematic mobile phone use, and disordered eating, there were no comprehensive investigations that explored their relationships. Our analysis suggested that mobile phone addiction contributes to the relationship between sleep and eating disorders. Previous studies have established that PSU is aggravated by daytime sleepiness, poor sleep quality and insomnia, which are in turn associated with impulsivity and poor self-regulation leading to higher risks of addiction (54, 55). PSU could be related to eating disorders in several ways. Distractions from excessive mobile phone use may impact satiety registration due to effects on the inferior frontal gyrus under distractive environments, leading to subconscious increased food intake (56). Bombardment of unrealistic body images, thin ideas and diet may incites stress and frustration in young adults (57). The compelling drive to adhere to the thin ideal also triggers body dissatisfaction, exacerbating risks of disordered eating (58, 59). Unbalanced time-allocation toward phone use and meals is another explanation that may result in meal skips, thus promoting unhealthy snacks (26) and ultimately eating problems.

In line with previous studies, psychological distress such as anxiety and depression symptoms significantly mediated the effect of sleep quality on disordered eating in the current study. Therese E. et al., found that depression and anxiety mediated the association between insomnia symptom severity and binge eating frequency (15). Selena et al., found that the relationship between sleep onset latency and emotional eating was mediated by trait anxiety but not depressive symptomatology in minority children (37). In a longitudinal study of clinical patients, depression was found to significantly mediates the relationship between poor sleep at admission (T0) and eating disorder symptoms after 6 months of standard treatment (T1) (36). In addition, the relationship between insomnia and eating psychopathology can be explained by both depression and anxiety in college women (9). This is the first time the roles of depression/anxiety in the relationship between sleep quality and disordered eating was explored for China Tibet college students including Tibetan, Han and other ethnic groups. In view of the complexity and multidimensional nature of sleep problems (60) and disordered eating (1), it is necessary to conduct further research into the mechanisms of psychological distress in the relationship between different sleep components and eating disorder subtypes.

We were able to confirm the direct effects of PSU on depression and anxiety that are consistent with previous reports (38, 61, 62). The serial mediation role we identified for PSU and psychological distress in the relationship between sleep quality and DEBs was “sleep quality -> PSU -> psychological distress -> DEBs.” Young adults who reported poor sleep quality tended to have higher levels of PSU and depression/anxiety symptoms, which in turn would lead to high levels of DEBs. This complex mediation pathway builds upon previous research (9, 15, 23, 36, 37), showing that psychology and behavior factors together contribute to the mechanism in the relationship between poor sleep and disordered eating.

These findings have important theoretical implications for understanding the development and prevention of DEBs. In theory, based on the sleep-health framework conceptualized by Buysee (60), sleep as a biological drive is bidirectionally related to physical, mental, and neurobehavioral health. This study extends the previous research by including a potential behavioral addiction and psychological distress (depression and anxiety symptoms) as intermediary variables to comprehensively explore their mechanism underlying how sleep quality exerts an effect on DEBs (14). On the other hand, from the perspective of practical implications, our model shows that it could be postulated that reducing levels of mobile phone addiction, anxiety and depression by improving sleep quality may be beneficial to students' disordered eating behaviors. Sleep hygiene, mobile phone and internet use hygiene, mental health education courses, professional psychological counseling and other interventions should be considered and implemented.

The current study has several interpretive caveats. First, the current study proposes a preliminary exploration for the associations, where longitudinal studies are greatly needed to further assess the causal relationship. Second, the data was collected by self-reported measures, so reporting bias may be introduced. Nonetheless, self-reported questionnaires were proven to be valid and applied worldwide. Third, although a number of potential confounders were included, there are some unmeasured confounders (e.g., parenting styles, substance use and other variables) that may contribute to these associations (63, 64). Forth, the current study only includes college students who are currently on campus and did not account for those absent. Despite its limitations, this study uses a relatively large sample to explore how mediations are related using multiple series mediation models, which expands the psychological and behavioral mechanisms of sleep problems affecting eating disorders.

## Conclusion

To conclude, this study comprehensively tested psychological and behavioral mechanisms underlying the association between sleep quality and disordered eating behaviors among university students from Tibet, China. Other than the direct effect of sleep quality on disordered eating behaviors, indirect pathways were clarified where sleep quality effects disordered eating behaviors through problematic smartphone use and psychological distress (i.e., depression/anxiety). Our research indicated that appropriated interventions that target problematic smartphone use could potentially reduce anxiety and depression level, which will in turn provide a buffer against the negative impact of poor sleep quality on eating disorder symptoms.

## Data Availability Statement

The raw data supporting the conclusions of this article are available through the Sun Yat-sen University. Contact Ciyong Lu for access approval.

## Ethics Statement

The studies involving human participants were reviewed and approved by Sun Yat-sen University, School of Public Health Institutional Review Board. The patients/participants provided their written informed consent to participate in this study.

## Author Contributions

RW: conceptualized and designed the present study, carried out the initial analyses, and drafted the manuscript. LG and CL: concept, design, and revising the manuscript. HR, JS, WL, MZ, and YH: design, formal analysis, and interpretation of the data and revising the article. WW and CL: project administration, supervising the project, and revising the manuscript. All authors have read and agreed to the published version of the manuscript.

## Funding

This work was supported by National Natural Science Foundation of China (International Cooperation and Exchange Programme: Grant No. 81761128030) and Natural Science Foundation of Tibet Autonomous Region [Grant No. XZ2018ZRG-83(Z)].

## Conflict of Interest

The authors declare that the research was conducted in the absence of any commercial or financial relationships that could be construed as a potential conflict of interest.

## Publisher's Note

All claims expressed in this article are solely those of the authors and do not necessarily represent those of their affiliated organizations, or those of the publisher, the editors and the reviewers. Any product that may be evaluated in this article, or claim that may be made by its manufacturer, is not guaranteed or endorsed by the publisher.
